# Insight on an Arginine Synthesis Metabolon from the Tetrameric Structure of Yeast Acetylglutamate Kinase

**DOI:** 10.1371/journal.pone.0034734

**Published:** 2012-04-18

**Authors:** Sergio de Cima, Fernando Gil-Ortiz, Marjolaine Crabeel, Ignacio Fita, Vicente Rubio

**Affiliations:** 1 Instituto de Biomedicina de Valencia del Consejo Superior de Investigaciones Científicas (IBV-CSIC), Centro de Investigación Biomédica en Red de Enfermedades Raras (CIBERER-ISCIII), Valencia, Spain; 2 Department of Genetics and Microbiology Emeritus, Vrije Universiteit, Brussel, Belgium; 3 Instituto de Biologia Molecular de Barcelona IBMB-CSIC/Institute of Research in Biomedicine (IRB-Barcelona), Parc Cientific, Barcelona, Spain; Helmholtz Centre for Infection Research, Germany

## Abstract

N-acetyl-L-glutamate kinase (NAGK) catalyzes the second, generally controlling, step of arginine biosynthesis. In yeasts, NAGK exists either alone or forming a metabolon with N-acetyl-L-glutamate synthase (NAGS), which catalyzes the first step and exists only within the metabolon. Yeast NAGK (yNAGK) has, in addition to the amino acid kinase (AAK) domain found in other NAGKs, a ∼150-residue C-terminal domain of unclear significance belonging to the DUF619 domain family. We deleted this domain, proving that it stabilizes yNAGK, slows catalysis and modulates feed-back inhibition by arginine. We determined the crystal structures of both the DUF619 domain-lacking yNAGK, ligand-free as well as complexed with acetylglutamate or acetylglutamate and arginine, and of complete mature yNAGK. While all other known arginine-inhibitable NAGKs are doughnut-like hexameric trimers of dimers of AAK domains, yNAGK has as central structure a flat tetramer formed by two dimers of AAK domains. These dimers differ from canonical AAK dimers in the −110° rotation of one subunit with respect to the other. In the hexameric enzymes, an N-terminal extension, found in all arginine-inhibitable NAGKs, forms a protruding helix that interlaces the dimers. In yNAGK, however, it conforms a two-helix platform that mediates interdimeric interactions. Arginine appears to freeze an open inactive AAK domain conformation. In the complete yNAGK structure, two pairs of DUF619 domains flank the AAK domain tetramer, providing a mechanism for the DUF619 domain modulatory functions. The DUF619 domain exhibits the histone acetyltransferase fold, resembling the catalytic domain of bacterial NAGS. However, the putative acetyl CoA site is blocked, explaining the lack of NAGS activity of yNAGK. We conclude that the tetrameric architecture is an adaptation to metabolon formation and propose an organization for this metabolon, suggesting that yNAGK may be a good model also for yeast and human NAGSs.

## Introduction

**Figure 1:**
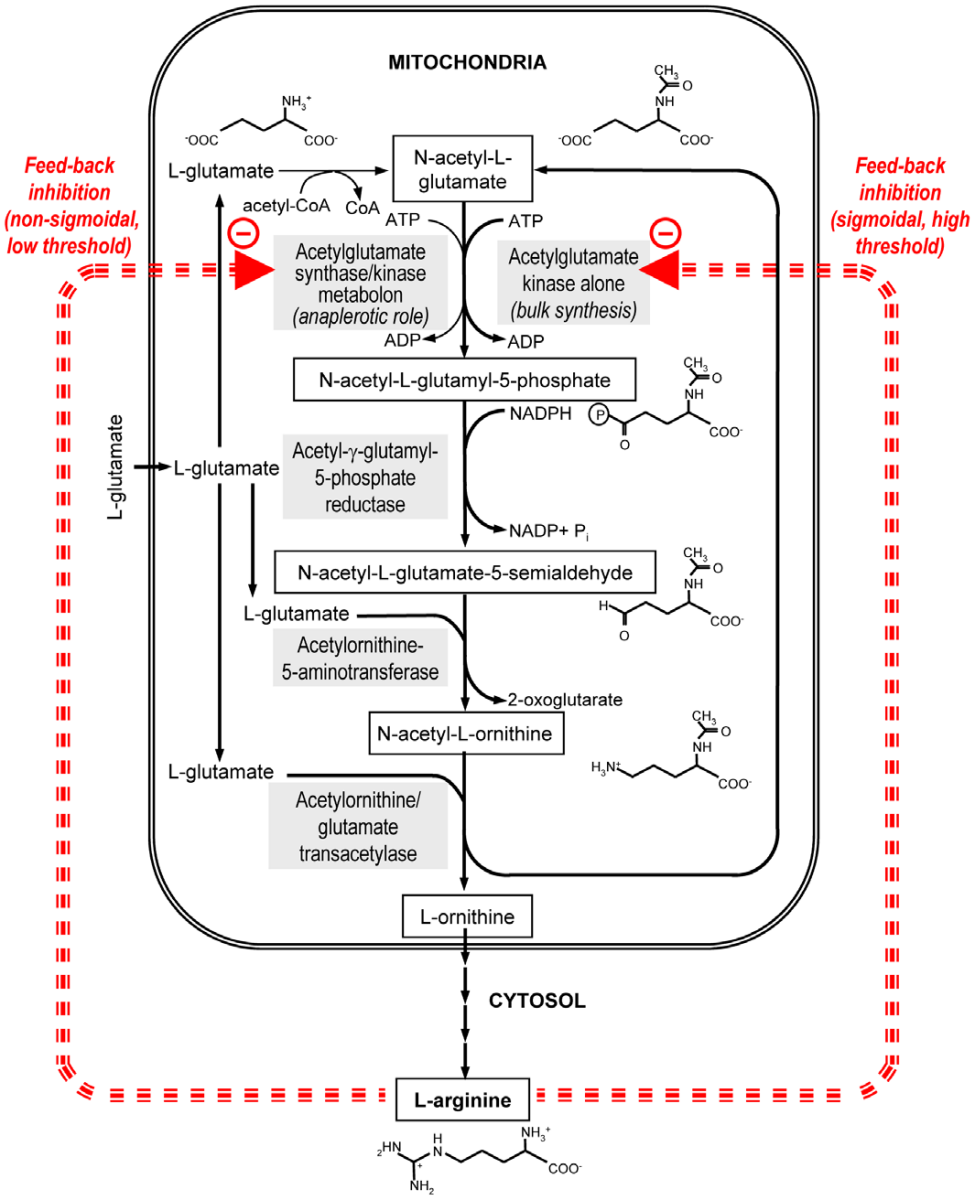
Arginine biosynthesis pathway of *Saccharomyces cerevisiae*. The kinase activity of the metabolon is inhibited by lower concentrations of arginine than N-acetyl-L-glutamate kinase alone. Red broken arrows indicate inhibition by arginine.

Microorganisms and plants make arginine from glutamate using a route in which intermediates are N-acetylated (Figure 1). The first step of the route is the production of N-acetyl-L-glutamate (NAG), while the second is the phosphorylation of NAG by NAG kinase (NAGK) [1], [2]. In some organisms like *Escherichia coli* this route is linear and NAG is made from acetyl CoA (AcCoA) and glutamate by NAG synthase (NAGS). However, many other organisms recycle the acetyl group by transacetylation from N-acetylornithine to glutamate (Figure 1), while the feedback control of the route by arginine is exerted at the NAGK level [1]–[3]. The regulatory importance of NAGK is highlighted by the fact that, in photosynthetic organisms, this enzyme is the target of P_II_, a signaling protein that orchestrates metabolic adaptations to nitrogen/carbon abundance [4], [5]. By binding to NAGK when ammonia is abundant, P_II_ relieves NAGK from arginine inhibition and allows nitrogen to stockpile as arginine [4], [5].

Because of the paramount regulatory role of NAGK, this enzyme has been the focal point of studies which have revealed the structures of the arginine-insensitive NAGK of *E. coli* [6] and of arginine-sensitive NAGKs from bacteria and from the plant *Arabidopsis thaliana* [7]–[9] either alone or complexed with P_II_. NAGK presents a typical subunit fold which is considered the paradigm of the amino acid kinase (AAK) family [6]. This fold consists of a characteristic α_3_β_8_α_4_ sandwich with a mostly parallel central β_8_-sheet, which is split into N- and C-lobes. All the NAGKs characterized structurally so far [6]–[9] are composed of identically shaped homodimers, in which the central β-sheet becomes continuous across the intersubunit junction due to contacts between the antiparallel β5 strands of the two subunits. Nevertheless, the structurally characterized NAGKs that are the subject of feedback inhibition by arginine have an extra N-terminal kinked α-helix, which links the NAGK dimers into doughnut-like hexamers with D3 symmetry [7]–[9]. Arginine binds at the interdimeric junctions next to these N-terminal helices and is believed to inhibit NAGK by expanding the hexameric ring, separating the two lobes of each subunit, thus preventing catalysis [7]–[9].

**Figure 2:**
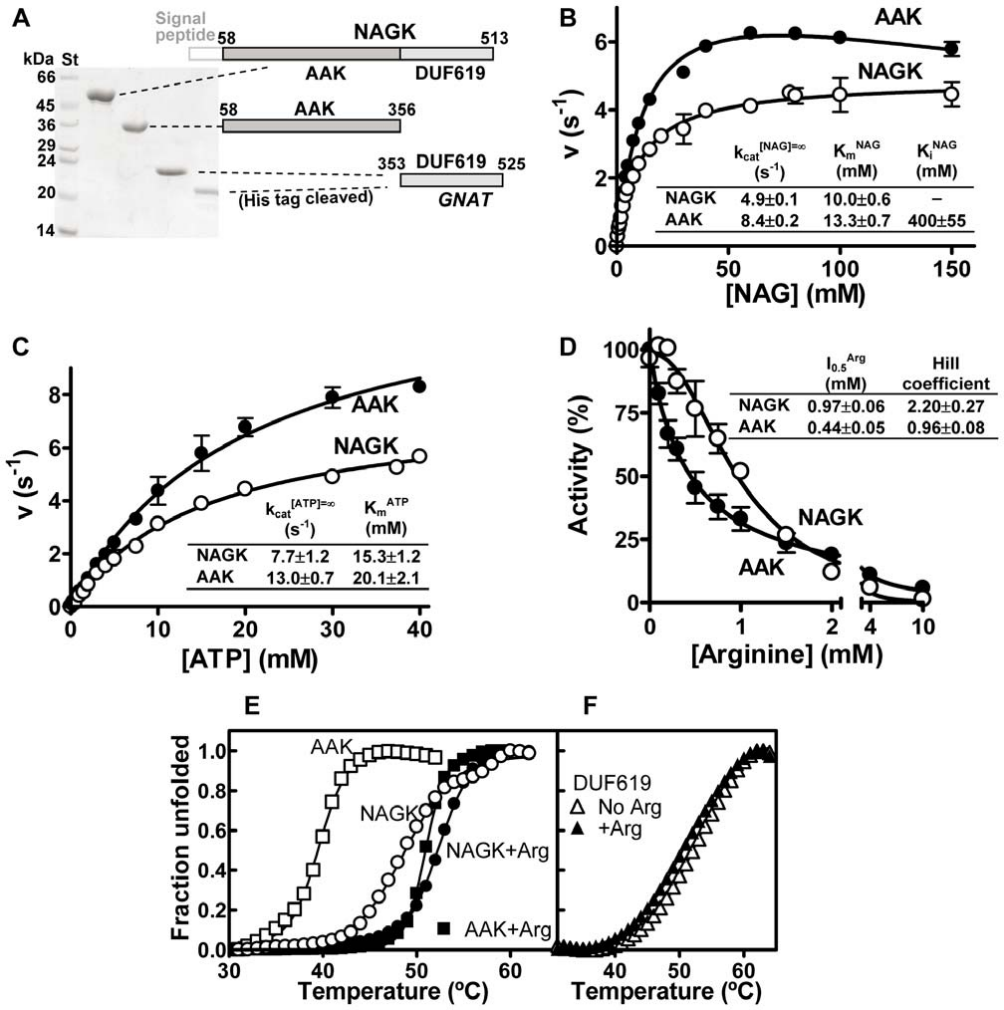
Yeast NAGK domain composition and effects of DUF619 domain deletion. (A) yNAGK domain composition and purification of the recombinant complete enzyme and its two isolated domains. *Left*, Coomassie-stained SDS-PAGE of the purified proteins. St; molecular markers, with masses given at the side. *Right*, bar representation of the domain composition of the whole enzyme and of the isolated domain constructs, with residue numbers. The mitochondrial signal peptide (faint tracing) was not included in the constructs. Both expressed domains are shown in different gray shades. For the isolated GNAT domain, the sequence was extended C-terminally to its potential maximum, corresponding to the beginning of the next enzyme encoded by the polyprotein precursor. The masses in kDa of the recombinant polypeptide chains, including their tags, are the following: yNAGK, 51; truncated AAK, 34; GNAT domain, without or after His-tag cleavage, 21.6 and 19.7 respectively. (B) and (C) Substrate kinetics for NAG and ATP. The results from the enzyme activity assays (see Materials and Methods; points are means ± standard errors (SE) for at least three determinations) were fitted in the case of the data for ATP and NAG to hyperbolae, except for the NAG kinetics for truncated yNAGK (AAK), which was fitted to hyperbolic kinetics with substrate inhibition according to the equation [52]: 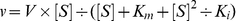. The inset tables provide means ± SE for the indicated parameters. k_cat_ values are given per polypeptide chain. (D) Arginine inhibition kinetics. The results were fitted to sigmoidal inhibition according to equation [3]: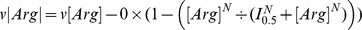, where *N* is the Hill coefficient and *I_0.5_* is the arginine concentration which gives 50% inhibition. The inset table gives the *I_0.5_* and *N* values (means ± SE). (E) and (F) Thermofluor assays with NAGK and its isolated domains. When used, L-arginine was at a concentration of 50 mM. For further details, see Materials and Methods.

We report here structural and functional studies on the arginine-sensitive NAGK from *Saccharomyces cerevisiae* (yNAGK). This enzyme, which is produced in yeast mitochondria by proteolytic processing of the polyprotein precursor (Uniprot database code Q01217) encoded by the *ARG5,6* gene, has the unexplained peculiarity, which is shared by the NAGKs of other ascomycetes, of having, in addition to the AAK domain, a C-terminal domain of ∼150 residues of unknown structure and function (Figure 2A) [10]–[12]. The same type of domain is found at the C-terminus in the NAGSs of ascomycetes and animals, including humans [13], [14]. This domain belongs to the DUF619 domain family (where DUF stands for domain of unknown function) of the Pfam (http://pfam.sanger.ac.uk) and InterPro (http://www.ebi.ac.uk/interpro) databases. Another very interesting characteristic of yNAGK is that it forms with yeast NAGS (yNAGS) a complex with the features of a true metabolon [12], [15], with its NAGS and NAGK components mutually influencing their regulatory properties by arginine [15] (Figure 1). Furthermore, whereas yNAGK can exist independently of yNAGS, the latter enzyme can exist only as a part of the NAGK/NAGS metabolon [12], [15]. Thus, yNAGK may also act as a chaperone and/or an essential stabilizing agent for yNAGS. Since ascomycetal NAGK and NAGS are homologous enzymes, which apparently exhibit the same domain organization [14], the ascomycetal NAGK/NAGS metabolon may have evolved from an ancestral oligomer formed by a single bifunctional enzyme molecule displaying both NAGK and NAGS activities. Such a bifunctional enzyme has been recently identified in *Xanthomonas campestris* [14].

To gain insight into the structural and regulatory complexities of ascomycetal NAGKs, we carried out *in vitro* expression and crystallographic studies of yNAGK. We demonstrated a modulatory and stabilizing role of the DUF619 domain on yNAGK. The structure of the mature yNAGK, lacking the N-terminal mitochondrial targeting sequence (residues 1–57 [12]), reveals an unexpected novel tetrameric architecture, which can be described as a dimer of dimers with D2 symmetry. The AAK domains occupy the center of the molecule flanked by two pairs of interacting DUF619 domains. A crystal, in which an arginine molecule is bound to one subunit, gives a snapshot of arginine-complexed and arginine-free subunits, which supports the previously proposed mechanism of arginine inhibition [7], [9]. The structure of the DUF619 domain reveals a histone acetyltransferase fold (GNAT domain fold) [16] in which the site for AcCoA is blocked. The yNAGK structure might help model that of human NAGS, an enzyme deficiencies in which cause inherited clinical hyperammonemia [13]. The structure also allows to propose a model for the NAGK/NAGS complex forming the yeast metabolon.

## Results and Discussion

### Roles of the DUF619 domain in yNAGK

A truncated form of yNAGK spanning from residue 58 to 356, corresponding to the AAK domain and lacking the C-terminal DUF619 domain, was produced as a soluble protein in milligram amounts in *E. coli* (see Materials and Methods and Figure 2A). Deletion of the DUF619 domain did not impair activity or arginine inhibition, as expected from the absence of a similar domain in bacterial and plant NAGKs [6]–[9]. In fact, the truncated yNAGK was even more active than the complete enzyme, as the deletion almost doubled the apparent k_cat_, whereas the K_m_ values for the two substrates, NAG and ATP, were little affected (Figures 2B and 2C). Thus, the binding sites for both substrates and the active center itself sit entirely within the AAK domain of yNAGK, while the presence of the DUF619 domain somewhat inhibits catalysis. In the truncated protein, the sensitivity to arginine is increased (Figure 2D), since the arginine concentration causing 50% inhibition (*I_0.5_*) was lowered, while the dependency of activity on arginine concentration was no longer sigmoidal. The sigmoidal nature of the arginine inhibition of the complete enzyme is an important attribute, making the enzyme little sensitive to low arginine concentrations but allowing the inhibition to escalate rapidly when the arginine concentration exceeds a given threshold. Therefore, the DUF619 domain exerts a modulatory effect on arginine inhibition which may prove important *in vivo*. Interestingly, the effects of DUF619 deletion on arginine inhibition kinetics replicate those observed when NAGK associates with NAGS in the NAGK/NAGS metabolon [15] (Figure 1), suggesting that the DUF619 domain is prevented from modulating NAGK inhibition in the metabolon.

An important stabilizing role of the DUF619 domain has been demonstrated in thermofluor unfolding studies [17] (Figures 2E and 2F). In these assays, the isolated DUF619 domain, produced recombinantly (Figure 2A), was approximately as stable as the complete enzyme (T_0.5_ values of 52°C and 49°C, respectively), whereas the truncation of the enzyme decreased its thermal stability by ∼10°C. The results also revealed a stabilizing effect, by approximately 4°C, of arginine on the complete enzyme, but particularly on the isolated AAK domain (T_0.5_ values of 39°C and 51°C in the absence and the presence of arginine, respectively), to the extent that the unfolding of this domain became virtually indistinguishable from that of the complete enzyme when arginine was present (Figure 2E). In contrast, arginine had no stabilizing effect (Figure 2F) on the DUF619 domain, thus confirming that the binding site for arginine is located in the AAK domain.

### yNAGK and its isolated DUF619 domain lack NAGS activity

The bifunctional NAGS-NAGK of *X. campestris* has been reported [14] to have the same domain organization as yNAGK, with both enzymes exhibiting comparable NAGK activities (1.95 and 3.31 U/mg, respectively). However, while the *X. campestris* enzyme is a very active NAGS having a specific activity of 76 U/mg, we did not detect (detection limit, <0.2 U/mg) any NAGS activity [18] with yNAGK in either pH 7 or 9, which is considered optimal for NAGSs, or in the presence or absence of MgATP, addressing the possibility of strict coupling between the potential NAGS activity and the NAGK activity. In contrast, the yeast NAGS/NAGK complex (data not shown) or the NAGS from *Pseudomonas aeruginosa* were very active NAGSs, having activities of 250 U/mg (pH 9, 37°C) or 79 U/mg (pH 9, 37°C [18]), respectively.

Recently, NAGS activity has been demonstrated with an NAGS of *Mycobacterium tuberculosis* [19] consisting exclusively of an isolated acetyltransferase domain that resembles the C-terminal domain of classical bacterial NAGSs [20], which is also similar to the DUF619 domain of yNAGK (see below). Thus, it is conceivable that the potential NAGS activity of the DUF619 domain of yNAGK might be repressed by the presence of the AAK domain, and that this domain in the NAGK/NAGS metabolon might be released from inhibition. However, constructs of the isolated DUF619 domain, with or without a His-tag (Figure 2A), did not exhibit even traces of NAGS activity at either pH 7 or pH 9 (detection limit, 0.1 U/mg). In contrast, and as a control of the assay, activity was observed with the NAGS from *M. tuberculosis* (1.5 U/mg at 0.1 M L-glutamate, pH 9; unpublished data from ML. Fernández-Murga in our laboratory). In summary, and in agreement with prior genetic evidence (reviewed in [12]) and with the results of assays in crude mixtures [15], yNAGK does not appear to be a bifunctional NAGK/NAGS.

### Structure of truncated yNAGK

**Figure 3:**
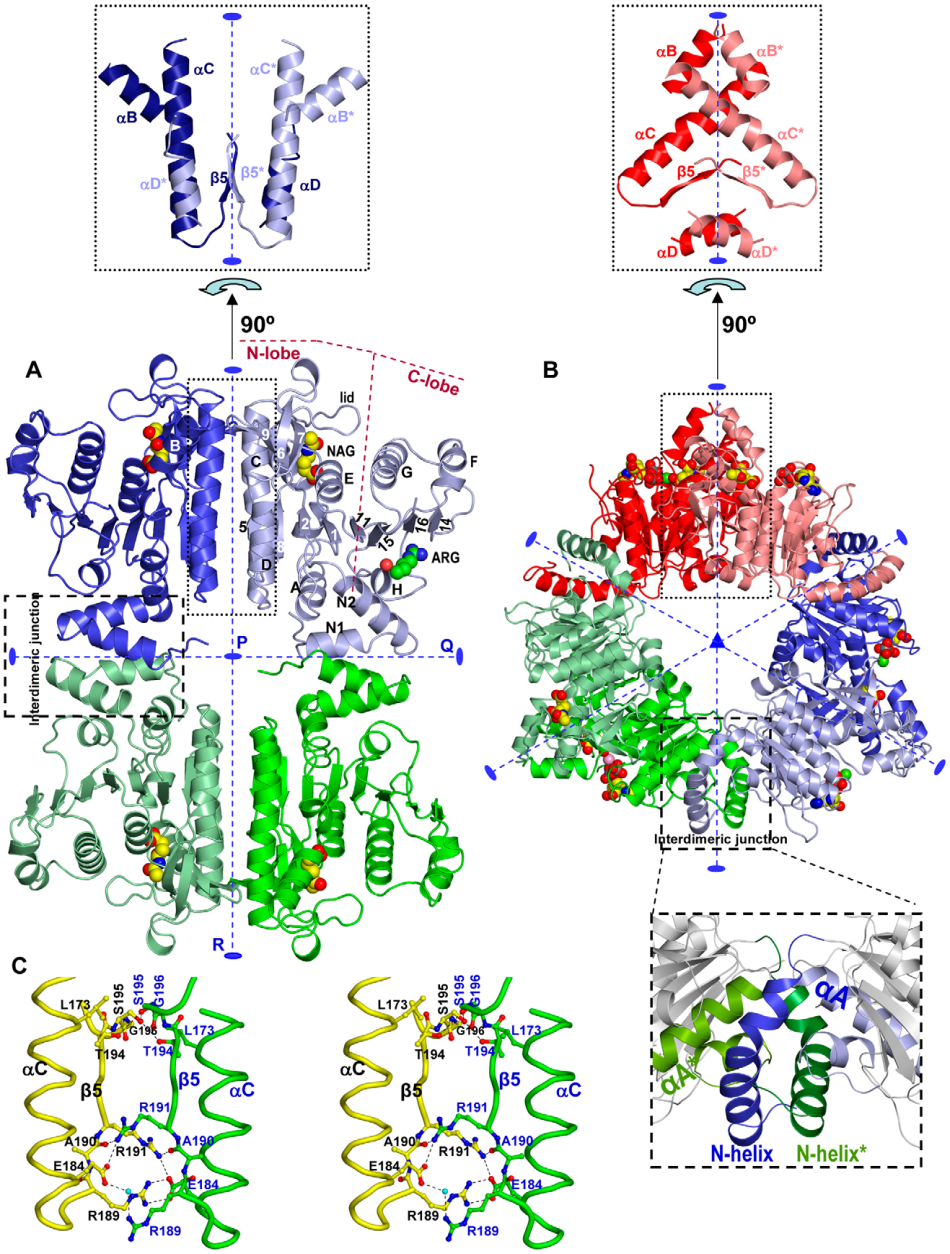
Structure of yNAGK lacking its DUF619 domain. Cartoon representation of (A) the tetramer of truncated yNAGK, as observed in the crystal soaked with arginine (2.1 Å resolution); and (B), of the hexamer of *P. aeruginosa* NAGK (PDB file 2BUF [7]). Two different hues of blue, green or red were used to identify the two subunits forming each R dimer. NAG and arginine in (A), and NAG and ADP-Mg in (B) are shown in space-filling representation. In (A) the N- and C-lobes and the secondary structure elements are labeled (helices with letters, strands with numbers) in the arginine-containing subunit, except helix B, which is labeled in the left-hand adjacent subunit. The 2-fold axes (labeled in (A) as P, Q and R) are shown as broken blue lines ending in blue ellipses, and the 3-fold axis of hexameric NAGK (B) is shown as a blue triangle. Interdimeric junctions are labeled and enclosed in broken-line rectangles in both (A) and (B). One such junction for the hexameric enzyme is enlarged in the lower inset of (B). In turn, the intersubunit junctions in the R dimer are enclosed in dotted-line rectangles, and they are illustrated in the top insets for (A) and (B), after 90° rotation around the R axes, relative to the main figures 3A and 3B. In the insets, elements from the two subunits are differentiated by the color code and also by placing asterisks on the labeled elements of one subunit. (C) Stereo view of the intersubunit bonds in the R dimer involving the β5 strands and the C-terminal moiety of helix C. This figure illustrates the β-sheet continuity across the intersubunit interface through side-chain bonds. Elements from one subunit are yellow and those from the other are green.

**Figure 4:**
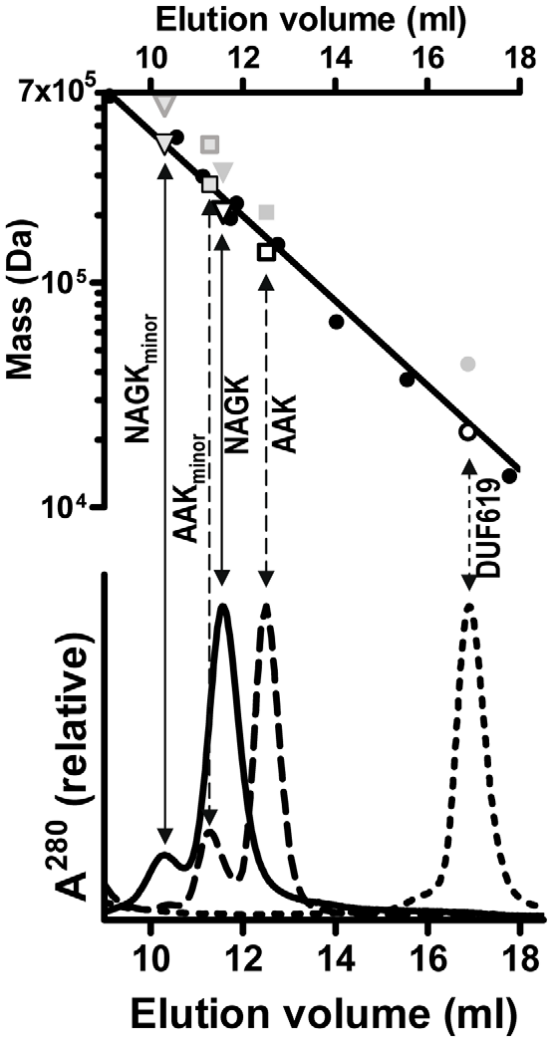
Size-exclusion chromatography of the complete enzyme and its isolated domains. The bottom panel illustrates the optical absorption (280 nm) of the effluent after injection to the Superdex 200 column of either complete NAGK (continuous line), AAK domain (broken line) and DUF619 domain (dotted line). The top panel illustrates a semi-logarithmic plot of molecular mass versus elution volume. Filled black circles correspond to the following protein standards (mass is given in kDa between parentheses): thyroglobulin (669), ferritin (440), NAGS from *P. aeruginosa* (294), β-amylase (224), NAGK from *P. aeruginosa* (191), alcohol dehydrogenase tetramer (147 kDa), bovine serum albumin (66), alcohol dehydrogenase monomer (37) and ribonuclease (14). Open-rimmed symbols plot the volumes of elution of the main peak for NAGK (inverted triangle) and for its AAK domain (square) versus their sequence-deduced masses if they were homotetramers, and for the DUF619 domain (circle) if it were monomeric (sequence-deduced masses 204, 132 and 21 kDa, respectively). Non-rimmed gray-filled symbols plot these positions for the sequence-deduced masses if NAGK and AAK were hexamers and if DUF619 were a dimer. Rimmed gray symbols correspond to the minor early peaks observed in the elution of NAGK and the AAK domain, by considering that both are octamers (black-rimmed) or dodecamers (gray-rimmed).

Four crystal structures of truncated yNAGK, ligand-free or complexed with either NAG or NAG and arginine, were determined using for the initial phasing single anomalous diffraction (SAD) from a Se-Met derivative (Table 1). Four subunits, organized as a dimer of dimers with D2 local symmetry, were found in the asymmetric unit of these crystals (Tables 1, 2 and Figure 3A). Given the fact that prior structures of arginine-sensitive NAGKs correspond to D3 hexamers (Figure 3B) [7]–[9], a tetrameric architecture was unexpected, but it was supported by size exclusion chromatography of both the truncated and the complete mature yNAGK proteins. The elution profiles of these proteins were more consistent with tetramers than hexamers (Figure 4). The small peaks observed to elute earlier with both the truncated and the complete yNAGK were consistent with octamers (possibly aggregates of two tetramers) rather than with dodecamers (aggregates of two hexamers) (Figure 4).

**Table 1:** Crystallographic data collection.*

Parameter	yNAGK lacking its C-terminal domain				Complete yNAGK		
	Se-Met-substituted	NAG-containing	Arginine-soaked	No ligands	Crystal 1^a^	Crystal 2^a^	Crystal 3
Beamline ESRF	ID14-4	ID14-4	BM16	ID14-4	ID14-4	ID14-4	ID23-2
Wavelength (Å)	0.9790	0.9393	0.9786	0.9393	0.9393	0.9393	0.8726
Space group	P2_1_2_1_2_1_	P2_1_2_1_2_1_	P2_1_2_1_2_1_	P2_1_2_1_2_1_	C2	P1	P1
Unit cell parameters							
a (Å)	69.8	69.7	69.5	68.1	112.0	95.2	92.3
b (Å)	99.2	99.3	99.6	100.5	146.7	111.3	103.3
c (Å)	189.8	190.6	189.2	188.7	70.7	113.1	111.3
α (deg)	90.0	90.0	90.0	90.0	90.0	75.8	77.3
β (deg)	90.0	90.0	90.0	90.0	101.4	89.3	89.3
γ (deg)	90.0	90.0	90.0	90.0	90.0	69.1	70.4
Solvent (%)	49	49	49	48	56	53	49
Resolution range (Å)	87.7–3.4	20–2.2	20–2.1	20–2.95	87.9–3.1	109–3.8	108–3.25
	(3.6–3.4)	(2.3–2.2)	(2.2–2.1)	(3.1–2.95)	(3.3–3.1)	(4.0–3.8)	(3.43–3.25)
Total reflections	216123	1015192	372634	178140	46836	94503	109862
Unique reflections	18793	67865	77012	28023	19757	39773	57473
I/σ_I_	4.9 (2.7)	5.0 (1.9)	3.8 (1.9)	8.3 (2.1)	9.3 (2.0)	10.5 (1.5)	9.6 (1.7)
R_sym_ b (%)	13.2 (24.8)	12.0 (40.5)	13.1 (36.7)	6.8 (37.3)	6.3 (34.7)	5.7 (45.5)	7.0 (45.6)
Completeness (%)	100 (100)	100 (100)	99.6 (99.6)	99.8 (99.8)	97.6 (97.6)	96.3 (96.3)	96.2 (96.2)
Wilson B factor (Å^2^)	41.5	26.7	19.8	79.7	86.8	97.4	72.2
Multiplicity	11.5 (11.9)	15.0 (15.3)	4.8 (4.7)	6.4 (6.5)	2.4 (2.4)	2.4 (2.4)	1.9 (1.9)
Phasing statistics for Se-Met substit.							
Se atoms	20	-	-	-	-	-	-
FOM^c^	0.762	-	-	-	-	-	-
CC all/weak^d^ (%)	43.1/21.2	-	-	-	-	-	-

* Values in parentheses are the data for the highest resolution shell.

a Data ellipsoidal truncation and anisotropic scaling were applied using the UCLA MBI Diffraction Anisotropy Server [54] to correct for strong anisotropy.

b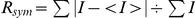, where *I* is the observed intensity, and *<I>* is the average intensity of the multiple observations of symmetry-related reflections.

c Figure of merit after density modification.

d Correlation coefficient all/weak as defined by program SHELX.

**Table 2:** Refinement statistics.

	yNAGK lacking its C-terminal domain			Complete mature yNAGK	
	NAG-containing	Arginine-soaked	No ligands	Crystal 2	Crystal 3
PDB code	3ZZF	3ZZH	3ZZG	3ZZI	4AB7
Resolution range (Å)	20–2.20	20–2.10	20–2.95	65.95–3.80	108.3–3.25
Reflections, work/test	64245/3432	72907/3860	26458/1398	37381/1985	54576/2896
R_factor_,^a^ work/test (%)	17.87/21.81	18.04/21.54	21.19/24.52	19.72/23.59	19.27/23.76
Average B-factors (Å^2^)					
Protein atoms	26.5	22.3	81.4	127.6	93.3
NAG	23.1	14.4	-	-	72.7
Arginine	-	29.3	-	-	-
Hg	43.6	-	-	-	-
Water	14.5	13.7	-	-	-
Number of:					
Polypeptide chains	4	4	4	8	8
Protein atoms	9107	9059	8978	27248	26294
NAG molecules	4	4	-	-	2
Arginine molecules	-	1	-	-	-
Hg atoms	8	-	-	-	-
Water molecules	430	497	-	-	-
RMSD bond (Å)	0.008	0.007	0.009	0.016	0.014
RMSD angle (°)	1.04	1.02	1.08	1.79	1.76
Ramachandran plot^b^ (%)					
Most favored	94.1	94.0	91.3	87.8	85.7
Additionally allowed	5.2	5.3	7.8	11.9	13.3
Generously allowed	0.7	0.5	0.6	0.3	1.0
Disallowed	0.0	0.2	0.4	0.0	0.0

a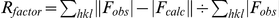 where *F_obs_* and *F_calc_* are the observed and calculated structure factors, respectively.

b Calculated using PROCHECK.

**Figure 5:**
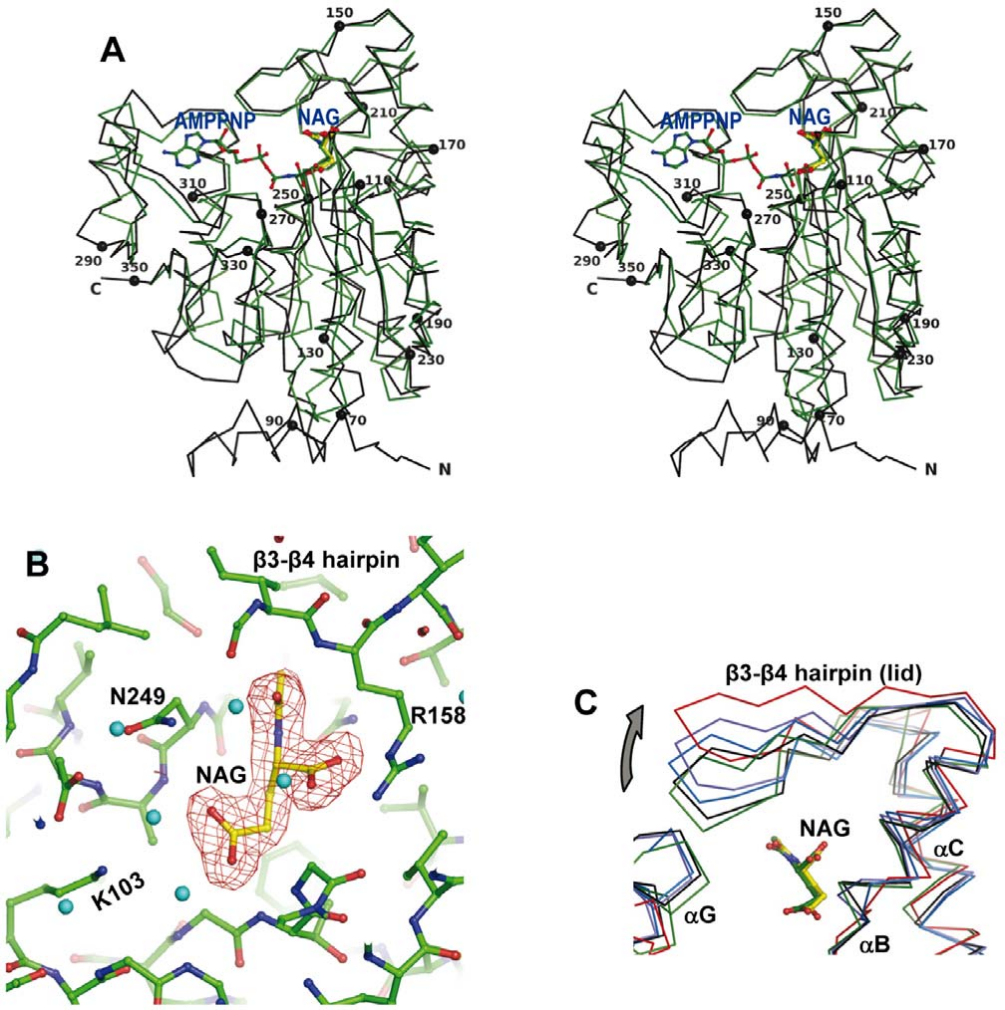
The yNAGK subunit and NAG binding. (A) Stereo view of the superimposition of backbone representation of EcNAGK (in green) with bound AMPPNP and NAG (ligands shown in green sticks), and of one truncated yNAGK subunit (2.2 Å resolution, black, with spheres marking every twentieth residue) bound to NAG (in yellow). (B) The NAG site. Detailed view of the substrate site of the NAG-containing truncated yNAGK crystal (2.2 Å resolution). The protein and ligands are shown in sticks representation, with the C atoms colored green in the protein and yellow in the ligands. The F_o_-F_c_ electron density map calculated omitting NAG from the model (omit map) is superimposed as a red grid and contoured at 3.0 σ. Cyan spheres are water molecules. Residues involved in NAG binding and the β3–β4 hairpin are labeled. (C) Backbone representation of the superimposition of the NAG site in two NAG-free (blue and violet) and one NAG-bound (black) subunits of truncated yNAGK, and in EcNAGK, either NAG-free (red) or bound to NAG and AMPPNP-bound (green) (*E. coli* structures taken from PDB codes 2WXB [21] and 1OHA [53], respectively), to illustrate NAG site lid mobility. Ligands (in sticks) have green-colored C atoms and yellow-colored ones for EcNAGK and yNAGK, respectively. Secondary structure elements are labeled.

Despite the different oligomerization of yNAGK in relation to other NAGKs, the AAK domain structure closely conforms to the canonic α_3_β_8_α_4_ sandwich NAGK subunit fold [6]–[9] (Figure 5A), with the N-lobe spanning residues 96–272 and the C-lobe residues 273–350. Even the lengths of the loops and of the secondary structure elements are similar to those of *E. coli* NAGK (EcNAGK) (root mean square deviation, r.m.s.d., 1.8 Å for superimposition of 246 Cα atoms). In the crystals containing NAG, one NAG molecule was found in each subunit (Figure 5B). As reported in other NAGKs (best studied in EcNAGK [6]), NAG is bound in a pocket in the N-lobe under the closed lid formed by the β3–β4 hairpin (Figures 5B and 5C). In the structure of the truncated yNAGK without NAG (resolution 2.95 Å, Tables 1, 2), the lid is observed in two different open conformations, which are, however, less open than in the structure of the NAG-free EcNAGK [21] (Figure 5C). Although no ATP-containing crystals were obtained, the ATP site is patent in the C-lobe at its expected location (Figure 5A) [6]. In fact, all the residues considered important for ATP binding and for catalysis are conserved as, for example, glycines 106, 137, 274 and 305, catalytic lysines 103 and 309, and the intervening aspartate 251, which correspond to EcNAGK glycines 11, 45, 184 and 213, lysines 8 and 217, and aspartate 162, respectively [6]. In summary, the subunit core fold, the substrate binding sites, the active center and the catalytic mechanism appear essentially identical in the AAK domain of yNAGK and in EcNAGK. In contrast with this high similarity of the AAK domains, the three possible dimers which could be defined in yNAGK by the three molecular axes PQR (Figure 3A) differ from those in other NAGKs. Nevertheless, the R dimer of yNAGK uses for dimer formation the same subunit surface, which is used by other NAGKs, although with a −110° relative rotation between the subunits around an axis perpendicular to the interacting surfaces (Figures 3A and 3B, top insets; and Figure S1). As already indicated, in canonic NAGKs the central β-sheet extends across the dimer due to the antiparallel pairing of the β5 strands of the two subunits (Figure 3B top inset). In yNAGK, the β5 strands of the two subunits still interact with each other, although they are parallel due to the large rotation of one subunit relative to the other (Figures 3A top inset, and 3C). Helices C and D, which sandwich β5, run parallel to the corresponding helices from the adjacent subunit, instead of forming the cross-grid observed with other NAGKs (Figures 3A and 3B top insets). Overall, the total occluded surface at the R interface, of 1044 Å^2^ per subunit as determined with the PISA server (see Methods), is divided into two approximately equal patches - one towards each end of helix C - leaving a non-interacting central region between these patches. As illustrated by the top dimer in the tetramer shown in Figure 3A, this R dimer of the yNAGK AAK domains has a butterfly-like shape and is flatter and more continuous around the molecular two-fold R axis than in canonic NAGKs.

Arginine-sensitive NAGKs, including yNAGK, have a 19–38 residue-long N-terminal extension which is responsible for joining the dimers into the corresponding oligomers [7]. In the hexameric NAGKs, this N-terminal extension (residues 1 to 25 in *P. aeruginosa* NAGK) consists of a protruding, slightly kinked helix that interlaces with the same helix of another subunit from a different dimer (Figure 3B and its bottom inset) [7]–[9]. However, in yNAGK the N-terminal extension comprises two helices forming an angle of approximately 45°, and a 5-residue N-terminal extended coil. These three elements form a platform or “stand” which mediates and determines the interactions between the dimers across the Q axis of the tetramer (Figure 3A, labeled “interdimeric junction”). At each interdimeric junction, a four-helix bundle is formed by both interacting “stands”, burying an approximate area of 910 Å^2^ per subunit. The bundle is stabilized mainly by hydrophobic interactions involving V71, I72, L75, V84, L88, and F91, but also by hydrogen bonds linking R68 and F91, and linking K81 with N76 and I78 (see Figure S2). Each “stand” is connected to the body of its cognate subunit by a large array of interactions, in particular with the C-ends of helices A and H and the adjacent loops, which are mainly hydrophobic and include several aromatic residues (Figures 3A and S2). This extensive network of interactions still allows conformational changes of one of the “stands” without junction dissociation, as when arginine was bound to one subunit (see below), and as reflected in the slightly different positions of the “stands” in different subunits. In any case, the interactions mediated by these stands result in the association of two yNAGK dimers into a relatively flat rectangular tetramer with approximate dimensions of 35 Å×109 Å×83 Å. This tetramer is perforated by a central hole of 18 Å diameter, which is formed between the two domain bodies and the two interdimeric junctions. Two active centers, separated by a distance of 80 Å, are exposed in one diagonal on each face of the tetramer (Fig. 3A).

### Structural changes triggered by arginine and the arginine inhibition mechanism

**Figure 6:**
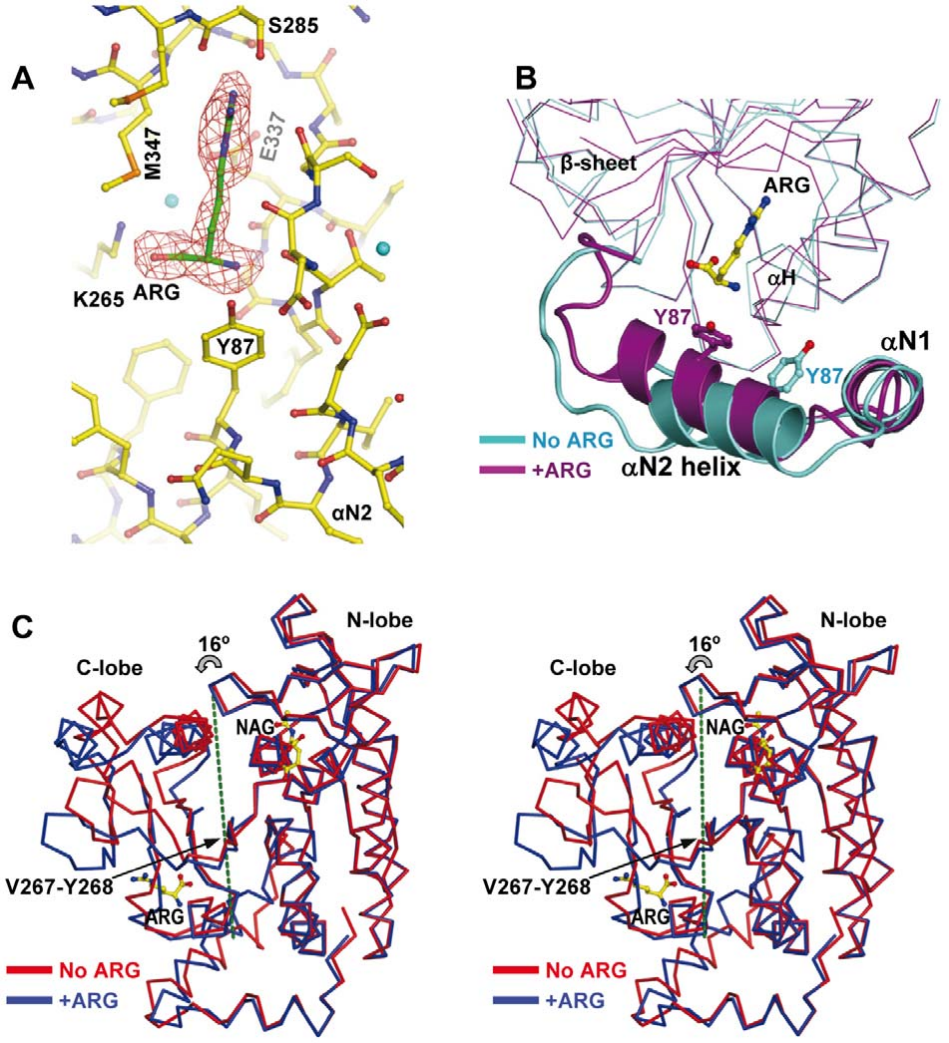
Arginine binding and associated conformational changes in the yNAGK subunit. (A) Arginine binding to truncated yNAGK (2.1 Å resolution). The protein and ligands are shown in sticks representation, with the C atoms in yellow in the protein, and in green in the ligand. The F_o_-F_c_ omit electron density map for arginine is superimposed as a red grid, contoured at 3.0 σ. This map was prepared by omitting arginine from the model in the refinement and calculation of the map. Cyan spheres are water molecules. Residues involved in arginine binding and the second N helix (αN2) are labeled. (B) Superimposition of the arginine-containing site (purple) with the arginine-free site (cyan) of another subunit to show second N-helix mobility. The C-lobe central β sheet, helix H, both N-terminal helices and the side chain of Y87 are labeled. The change in the position of the Y87 side-chain reflects the rotation of the second N-helix around its central axis (see the text). (C) Stereo view of the superimposition of the arginine-containing subunit (blue) and of an arginine-free subunit (red) of the same tetramer (subunits C and A, respectively), revealing the 16°-rotation of the C-lobe around the drawn axis (green broken line) and the indicated hinge residues. Bound NAG and arginine are shown in sticks. Domain orientation is the same as in Figure 3 of [21].

Whereas one normally bound NAG molecule was found sitting in each subunit of the yNAGK tetramer of the arginine-soaked crystal (resolution, 2.1 Å; Tables 1 and 2), only one subunit contained bound arginine. The arginine molecule sits where expected [7], [9], at the free end of the C-lobe, between the second N-helix, the central β sheet, helix H and the loop connecting this helix to β16 (Figures 3A, 6A and 6B). There are important conformational differences between the arginine-containing and the arginine-free subunits (Figure 6C). In the arginine-containing subunit the C-lobe of the AAK domain has moved away from the N-lobe, around a hinge located at the interlobar boundary (residues V267-Y268). This movement can be described as a 16° rigid-body rotation of the C-lobe about an axis that crosses obliquely the central β sheet at the level of β11 (Figure 6C). A similar open conformation has been reported in arginine-bound hexameric NAGKs [7], [9] and, with somewhat different characteristics, in substrate-free EcNAGK [21]. In the open conformation, the active center is distorted, with the sites for the two substrates being separated, thus likely corresponding to an inactive enzyme form [21]. Therefore, these results support the view that arginine, by binding to both the second N-helix and the C-lobe, pulls the C-lobe away from the N-lobe, which is firmly anchored to the other subunit of the R dimer, stabilizing the open inactive conformation of the AAK domain, thus causing the inhibition.

Arginine binding is also associated with structural changes in the second N-helix, which, in the arginine-free subunits, is dislodged from the arginine binding site by a displacement of 4.5 Å (maximum displacement of a Cα atom, 8 Å) and a rotation of 25° about its central axis (Figure 6B). These changes in the arginine binding site are not propagated across the interdimeric junction, which thus becomes nonsymmetric (Figure 3A), explaining the lack of cooperativity for arginine inhibition of truncated yNAGK (Figure 2D). Changes at the arginine binding site diminish the buried surface at the interdimeric junction of yNAGK from 910 Å^2^ per subunit in the arginine-lacking tetramers to 840 and 800 Å^2^ in the arginine-containing and arginine-free subunits, respectively. However, as the stability of the truncated enzyme is remarkably increased by arginine (Figure 2E), this diminished buried surface could somehow be artefactual due to having only one subunit with arginine and a broken symmetry.

### Structure of the complete mature yNAGK

**Figure 7:**
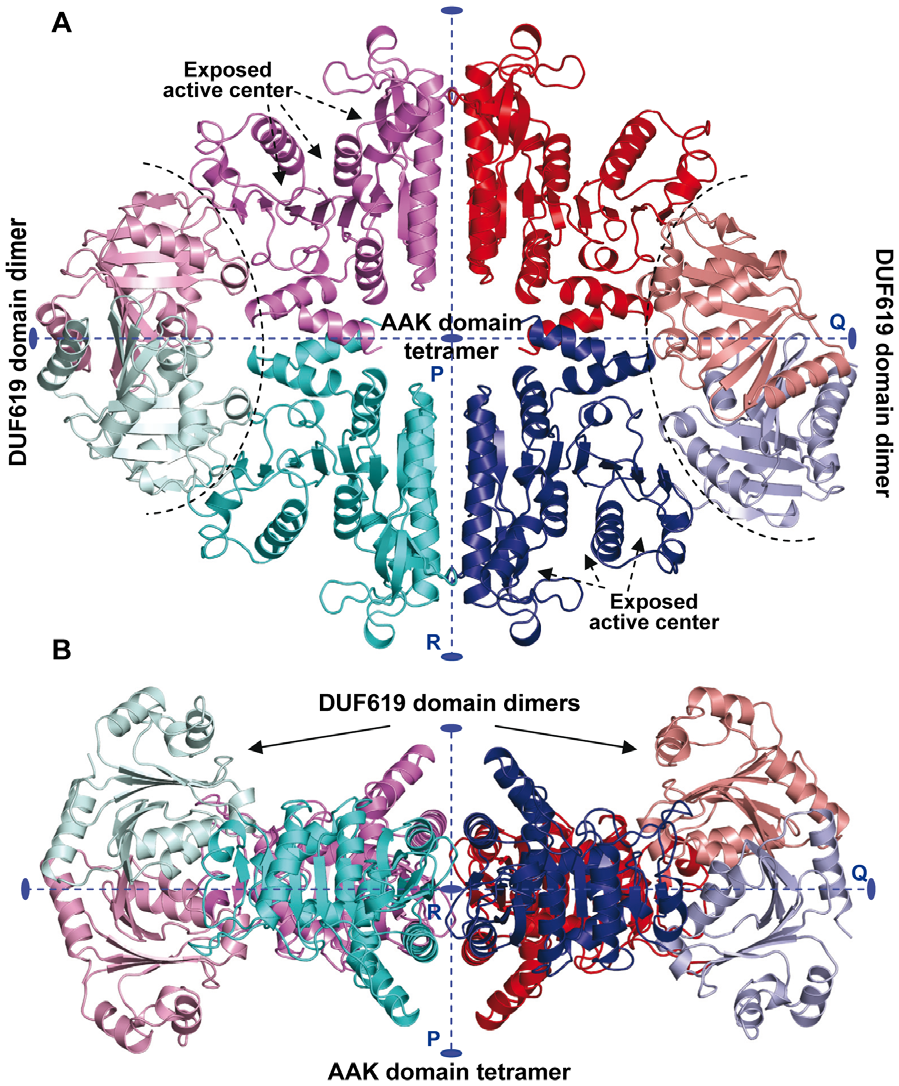
Structure of complete mature yNAGK. Cartoon representation of (A) front and (B) side views of the complete enzyme. The side view corresponds, in relation to the front view, to a 90°-rotation along the Q axis. In both views, one dimer is red and magenta and the other blue and cyan, and the AAK and DUF619 domains of each subunit are in different hues of the same color. In the front view, the two exposed active centers on one side of the molecule are labeled and marked with arrows, and curved broken lines separate the DUF619 domain dimers from the AAK domain tetramer. The 2-fold axes (labeled as P, Q and R) are shown in both figures.

**Figure 8:**
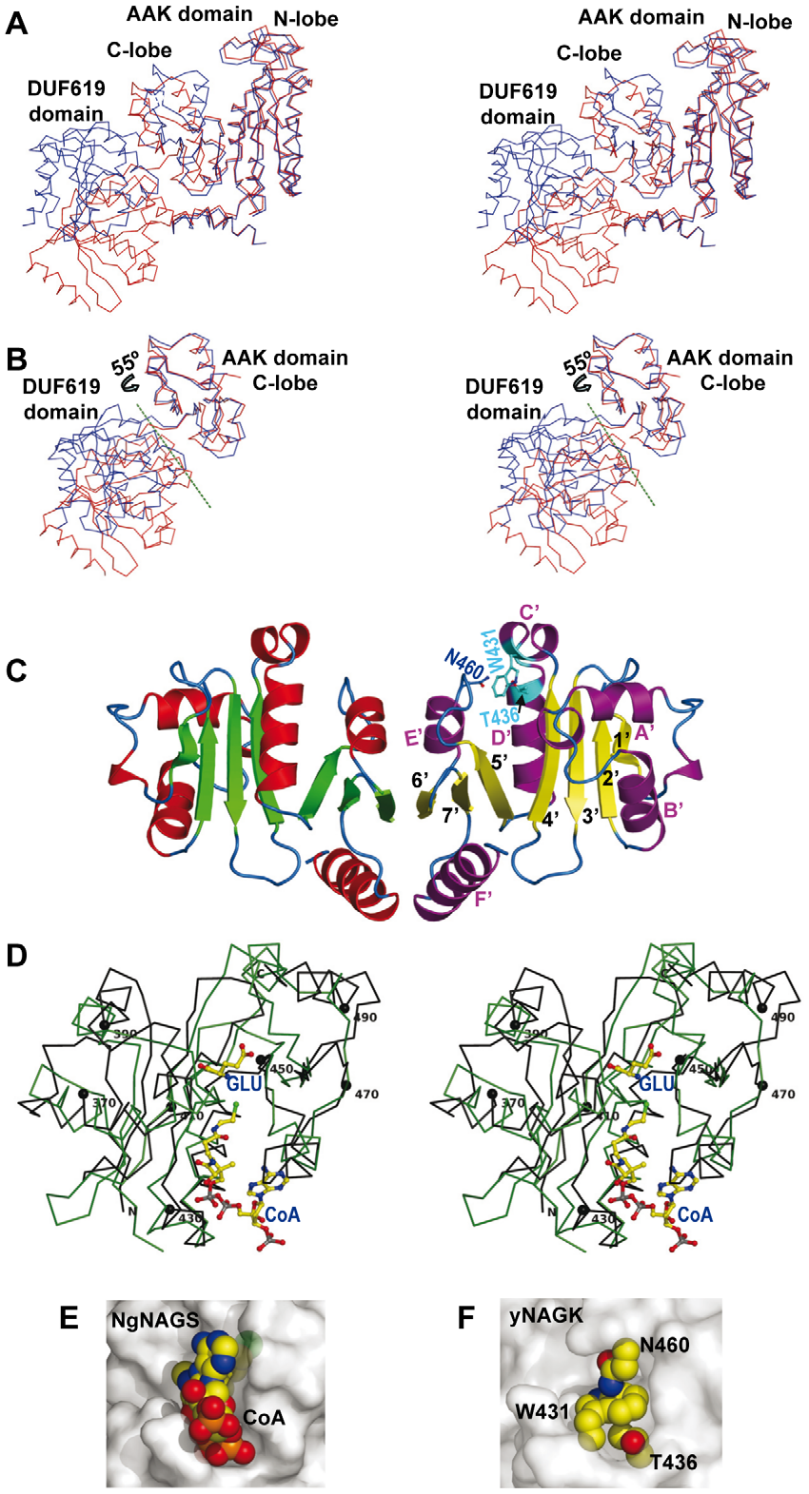
The DUF619 domain. (A) and (B) Stereo views to show movements of the AAK domain C-lobe and GNAT domain. In (A), the N-lobes of an open (red) and a closed (blue) subunit of the complete enzyme are superimposed, whereas in (B), the AAK-domain N-lobe is not shown and the C-lobes of this domain of both subunits are superimposed, illustrating the 55°-rotation associated with subunit opening (red), with the axis of the rotation shown as a green broken line. (C) The DUF619 domain dimer. Each subunit is in a different color (α helices red or magenta, β sheets yellow or green, with loops in blue in both subunits). Secondary structure elements are labeled in the subunit on the right. The side-chains of W431, T436 and N460 (in cyan or blue), which block the AcCoA channel, are shown in this subunit and are labeled. (D) Superimposition of the backbone of the GNAT domains of yNAGK (black, with every twentieth residue marked with a sphere and labeled) and of NgNAGS (green) (PDB code 3D2M [22]). The CoA and glutamate, found in the latter structure, are illustrated in sticks representation with C atoms in yellow. (E) and (F) Comparison of the AcCoA site of NgNAGS (E) in surface representation, with bound CoA in spheres, and the corresponding region of yNAGK (F), in the same orientation, showing the side chains of W431, T436 and N460 in spheres representation to illustrate the blocking of the AcCoA channel by these side chains.

Two closely related, yet non-isomorphous crystals of full-length mature yNAGK, each containing two enzyme tetramers in the asymmetric unit, have allowed determination of the structure of complete yNAGK using X-ray diffraction (Tables 1 and 2; Protein Data Bank, PDB, (www.pdb.org) accession codes 3ZZI and 4AB7). Although the resolution attained was 3.25 Å at the most, the high quality of the averaged electron density map (illustrated in the Figure S3) allowed to unambiguously trace the entire polypeptide and to identify the majority of amino acid side chains, particularly for the DUF619 domain. The full-length enzyme tetramers consist of the AAK domains occupying the central part of the molecule, as in the truncated enzyme structure, flanked at both ends along the Q axis by a pair of interacting DUF619 domains (Figures 7A and 7B). Projected along the P axis, the enzyme presents an elliptic profile with minor (R) and major (Q) axes of 109 Å and 140 Å, respectively. The central hole described for the truncated enzyme is also present, together with two narrow openings between the DUF619 and the AAK domains (Figure 7A). The lines joining the centers of mass of mutually interacting DUF619 domains are oblique in relation to the flat AAK tetramer; thus DUF619 domain pairs protrude from the plane defined by the four AAK domains (Figure 7B). The structures of the four tetramers found in the two crystals deviate from perfect D2 molecular symmetry, with three subunits in each tetramer exhibiting the open AAK domain conformation. The degree of AAK domain opening is even higher than in the arginine-containing subunit of the truncated enzyme, corresponding to a 20° rotation of the C-lobe (Figure 8A). The pulling of the C-lobes by the paired DUF619 domains possibly contributes to this opening of the AAK domain, which is associated with rotations of up to 55° of the DUF619 domain around a hinge located at the AAK domain-DUF619 domain linker (^351^GYK) (Figure 8B). It is interesting that arginine inhibition of *Neisseria gonorrhoeae* NAGS (NgNAGS) is associated with a rotation of approximately 109° of the C-terminal domain of this enzyme around the linker connecting it to the AAK domain [22]. As shown below, this domain of NgNAGS corresponds to the DUF619 domain of yNAGS.

Comparison of the complete and truncated NAGK structures explains why the presence of the DUF619 domains does not influence the K_m_ values for the substrates. This lack of effect on the apparent affinity for both substrates reflects the lack of physical interactions of the DUF619 domains with the substrate sites, which remain equally exposed in the structures of the truncated (Figure 3A) and the complete enzymes (Figure 7A). In contrast, the increase in k_cat_ associated with the truncation parallels the increased tendency of the AAK domains of the complete enzyme, relative to the AAK domains of the truncated enzyme, to adopt the open inactive conformation. The decrease in the *I_0.5_* for arginine upon truncation may result from the loss of the contacts existing in the complete enzyme between the DUF619 domain and the arginine binding site of the same subunit. These contacts, which include interactions with the second N-helix and, in some subunits, with the mobile loop at the end of helix H (Figure 7A), may hamper the arginine binding site adopting a conformation that is competent for binding the feed-back inhibitor. The sigmoidal arginine inhibition kinetics of the complete enzyme suggests the propagation, mediated by de paired DUF619 domains, of the changes in one arginine binding site to the arginine binding site across the same interdimeric junction. In agreement with this view, the Hill coefficient for arginine inhibition kinetics for the complete enzyme is 2, whereas inhibition of the truncated enzyme is non-sigmoidal (Figure 2D). It is noteworthy that, in the NAGK/NAGS metabolon, these effects of the DUF619 domain on arginine regulation are lost [15]. Therefore, binding of yNAGS to yNAGK possibly abolishes the interactions between the NAGK DUF619 domains and the arginine binding sites, most likely because of structural rearrangements in the NAGK tetramer.

The much higher temperatures needed for unfolding the complete yNAGK than for unfolding the truncated yNAGK (Figure 2E) indicate structural stabilization by the presence of the DUF619 domains, perhaps due to the pairing of these domains and to their interactions with the AAK domains of their cognate subunits.

### The DUF619 domain presents the histone acetyltransferase fold

The DUF619 domain is a bilobar (N-moiety, residues 354–447; C-moiety, residues 448–502) open αβα sandwich nucleated by a 7-stranded antiparallel β-sheet, which has the characteristic architecture of the histone acetyltransferase fold (GNAT fold) (Figure 8C) [16]. This architecture is shared also by the acetyltransferase domain of NgNAGS (Figure 8D), the domain that in this enzyme binds glutamate and AcCoA and catalyzes acetyl transfer. It includes a typical V-shaped central sheet, in which strands 4′ and 5′ diverge, with strand topologies for the N- and C-moieties of the sheet, 1′2′3′4′ and 5′7′6′, respectively. The comparison of the GNAT domains from NgNAGS and yNAGK (with an r.m.s.d. of 2.52 Å for 111 residues) explains why yNAGK lacks NAGS activity. The AcCoA binding site in NgNAGS, found on the boundary of the N- and C-moieties, is blocked in yNAGK (compare Figures 8E and 8F). The CoA recognition motif R/Q-x-x-G-x-G/A [23], which is present in NgNAGS (^364^ QDGGYG ^369^) at the entrance of helix D′, is replaced in yNAGK by ^431^ WLNNVT ^436^ (the motif-characteristic residues are underlined) (Figure 8C). T436 replaces one glycine of the CoA binding motif. W431, sandwiched between N460 and T436, blocks the part of the site where the pyrophosphate moiety of the CoA molecule would be expected to bind, thus rendering impossible AcCoA binding (Figures 8C and 8F). In contrast, in the *X. campestris* bifunctional NAGS/NAGK enzyme, the CoA recognition signature, ^350^ QGEGLG, is preserved, therefore supporting the view that changes in the AcCoA site correlate with the lack of NAGS activity in yNAGK.

The pairing of the DUF619 domains on each side of the yNAGK tetramer involves hydrophobic interactions between the E′ and F′ helices as well as the continuity of the central β sheets by way of antiparallel bonding of strands 6′ from both domains (Figure 8C). These interactions are maintained in the four pairs of DUF619 domains observed in the present crystal structure (with r.m.s.d.s from 0.85 to 1.45 Å), despite the important departures from the D2 symmetry observed in the yNAGK tetramers. The contacts among the DUF619 domains, although not strong enough for dimer formation when the DUF619 domains are isolated, judging from the size exclusion chromatography results (Figure 4), could contribute ∼2800 Å^2^ of extra buried surface in the whole enzyme tetramer, and should, thus, substantially contribute to tetramer stability. The plasticity of the “stands” at the dimeric junctions, together with the rigidity of the pairing of the DUF619 domains, should restrain the mobility required by the opening/closing of the active centers in the AAK domains.

### Final remarks

**Figure 9:**
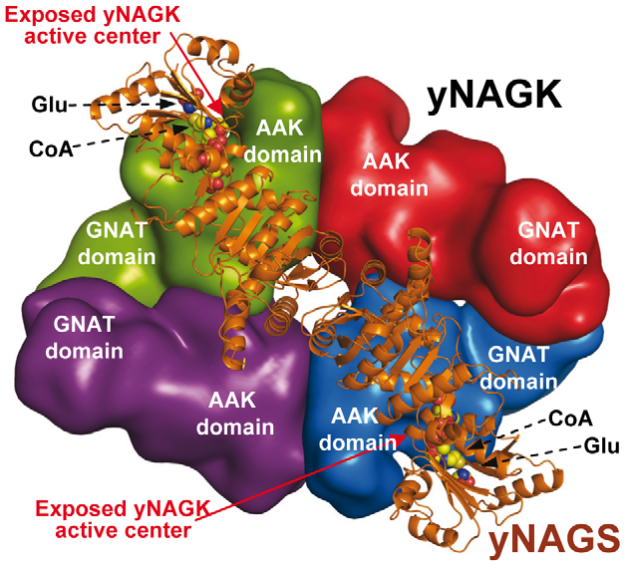
Model of the yeast NAGK/NAGS complex. Crude model of the proposed architecture of the complex of yNAGK and yNAGS. Given the similarity of yNAGK and yNAGS, the latter has been represented as one of the yNAGK dimers observed in the present structures, superimposing the CoA and glutamate observed in the structure of the corresponding domain of NgNAGS in the GNAT domain to illustrate the nearness of the NAG site to the active centers that are exposed on one side of the yNAGK tetramer (labeled). Since another dimer of the yNAGS dimer would sit on the other flat face of the yNAGK tetramer, the complex stoichiometry for complete occupation of yNAGK by yNAGS would be 4∶4 in terms of number of subunits of both proteins. No attempt has been made to model the interactions between the GNAT domains of both proteins.

The present work raises questions as to why yNAGK has an inactive GNAT domain and a distinctive tetrameric architecture. Identification of a bifunctional NAGS/NAGK enzyme in *X. campestris*, with apparently the same domain organization as yNAGK and a high NAGS activity [14], suggests that the yeast NAGK/NAGS metabolon could have evolved from an ancestral bifunctional NAGS/NAGK enzyme. Loss of NAGS activity in one component and of NAGK activity in the other may have arisen when a cyclic arginine biosynthetic route was introduced, downgrading the role of the NAGS activity to a merely replenishing (anaplerotic) one (Figure 1). In this context, it appears advantageous to separately regulate NAGS and NAGK levels by having independent genes for these two enzymes rather than having a single bifunctional NAGK/NAGS. To prevent deacylation of NAG by deacylases, which are abundant in animals [24] and perhaps also in ascomycetes, the yNAGK/yNAGS metabolon could serve to channel NAG from its production site in the GNAT domains of yNAGS to the phosphorylation sites in the AAK domains of yNAGK. The flat tetrameric nature of yNAGK, with the exposure of two active centers on each face of the tetramer, appears well suited for the association with yNAGS dimers, assuming that the structure of yNAGS is similar to the one determined here for yNAGK (Figure 9).

The present results may also be relevant for human NAGS, whose inborn errors cause clinical hyperammonemia [25]. The closeness of human and yeast NAGSs [26], [27] suggests that both share the domain composition of yNAGK [14], including the presence of the C-terminal DUF619 domain. Therefore, the view [25] that the hexameric structure of NgNAGS [20] may be a model for human NAGS should be re-evaluated. In fact, the few data available on mammalian NAGS suggest that this enzyme is a lower oligomer than hexamers [28], [29]. Nonetheless, the fact that yNAGS, with no independent existence of yNAGK, is the closest non animal homolog of human NAGS [14], [25], [26] renders it conceivable that human NAGS might be associated with other proteins. This may fit the low stability of the mammalian enzyme [29]. In this context, and given the difficulties to obtain crystalline mammalian NAGS, determination of the structure of the yeast NAGK/NAGS complex might provide insights into the structure of human NAGS. Therefore, efforts are being invested in our laboratory to ascertain the structure of the yeast NAGK/NAGS complex.

## Materials and Methods

### Production of yeast NAGK and its isolated AAK and DUF619 domains

To produce yNAGK which lacks the mitochondrial targeting sequence and is preceded by a MGH_6_ His-tag, the DNA sequence encoding enzyme residues 58–513 (see Table S1) was PCR-amplified from pYB3 [15] and inserted into the *NcoI-HindIII* sites of pTrc99a (from Pharmacia; now discontinued). yNAGK was truncated after residue 356 by replacing in the yNAGK-encoding plasmid codon 357 by the TGA stop codon, using the site-directed mutagenesis QuickChange kit (Stratagene), and primers 3 and 4 (Table S1). The DUF619 domain-encoding sequence was PCR-amplified (Table S1) from *Saccharomyces cerevisiae* (strain FY250) genomic DNA (a gift of P. Sanz, IBV-Valencia) and inserted at the *NheI/BamHI* sites of pET28a. This construct fuses the His-tag including a thrombin cleavage site, MGSSH_6_SSGLVPRGSHMAS, to residue 353 of yNAGK, which is the first of the DUF619 domain. All the constructs were verified by automated DNA sequencing.

The complete enzyme and its AAK domain were expressed in *E. coli* BL21 (DE3) grown to OD^600^ = 0.5–0.6 (37°C, aeration) in 0.5–1 L LB medium, supplemented with 0.1 mg/ml ampicillin and then induced for 20 hours at 30°C with 1 mM isopropyl-β-D-1-thiogalactopyranoside (IPTG). The Se-Met-substituted AAK domain was produced in the same way by replacing LB with SelenoMet Medium (Molecular Dimensions, Newmarket, UK), adding to this medium 15 min before IPTG induction an amino acid mixture containing 60 mg/L L-Se-methionine, 100 mg/L of each, L-lysine-HCl, L-phenylalanine and L-threonine, and 50 mg/L of each, L-isoleucine, L-valine and L-leucine [30]. The antibiotic used for the DUF619 domain expression was kanamycin (0.06 mg/ml), and the culture was placed for 30 min on ice before induction with 0.02 mM IPTG, continuing the culture for 20 hours at 15°C.

Subsequent steps were performed at 4°C. After centrifugation, cell pellets were sonicated in 1/25 of the original volume of a solution containing 20 mM Tris-HCl pH 7.5, 10 mM MgCl_2_, 1 mM dithiothreitol and either 0.25 M (complete yNAGK) or 0.5 M (AAK or DUF619 domains) NaCl, and the supernatant of the centrifuged sonicate was applied to a 1-ml HisTrap column fitted in an ÄKTA FPLC (from GE Healthcare). This column had been equilibrated with the sonication buffer enriched with 20 mM imidazole, and was washed successively with 5 ml of the same solution and then with 15 ml of 70 mM imidazole-containing solution. The pure proteins, eluted by increasing the imidazole concentration to 0.5 M, were concentrated to ∼10 mg/ml and freed from imidazole by centrifugal ultrafiltration.

The His-tag was removed from the DUF619 domain by overnight digestion at 4°C with agarose-inmobilized thrombin (Thrombin Clean Cleave Kit, Sigma, USA) in 50 mM Tris-HCl pH 8.0, 10 mM MgCl_2_, 10 mM CaCl_2_ and 0.25 M NaCl, collecting the tag-free protein by passing through a His-trap Ni-affinity column according to the kit instructions. Following confirmation of tag removal by SDS-PAGE (15% gels, Figure 2A), the protein was concentrated to ∼10 mg/ml and placed in sonication buffer for storage, as above.

For crystallization purposes, the surface-exposed lysine residues of the complete enzyme were reductively dimethylated [31] using a commercial kit (JBS Methylation Kit, from Jena Bioscience GmbH, Germany), following the manufacturer's instructions. Then the enzyme was concentrated to ∼10 mg/ml and was placed in 20 mM Hepes, pH 7.5, 1 mM β-mercaptoethanol and 0.5 M NaCl.

### Enzyme activity assays

NAGK activity was determined colorimetrically in the presence of 0.4 M hydroxylamine [32] at 37°C. When NAG was varied, ATP and MgCl_2_ were fixed at 20 mM and 30 mM, respectively. When ATP was varied, the NAG concentration was fixed at 80 mM and that of MgCl_2_ was kept in 10 mM excess over that of ATP.

NAGS activity was determined at 37°C as glutamate-triggered CoA release from AcCoA by monitoring colorimetrically SH group production with Ellman's reagent [18], [19]. The assay mixture contained 0.1 M Na L-glutamate, 4 mM AcCoA and either 0.2 M triethanolamine-HCl, pH 7.0 or 0.2 M Tris-HCl, pH 9.0. Whenever indicated, the assay mixture was supplemented with 20 mM ATP and 30 mM MgCl_2_. The reaction was initiated with the enzyme and was stopped after 30 min by a 40-fold dilution with 0.2 mM Ellman's reagent in 0.1 M Na phosphate, pH 7.0, determining absorbance at 412 nm.

### Crystallization and data collection

Table 1 summarizes the data on the different crystals. The sparse matrix vapor diffusion sampling approach [33] was followed in crystallization screens at both 21°C and 4°C, in 0.8 µl sitting drops (1/1 protein solution/reservoir fluid), either with or without additions of 24 mM NAG, 20 mM MgCl_2_, 6 mM of the inert ATP analog adenosine 5′-(β,γ-imido) triphosphate (AMPPNP) or 2 mM L-arginine-hydrochloride, added individually or as mixtures. The DUF619 domain, either with or without His-tag, yielded no crystals. The best crystals of the truncated enzyme (native or Se-Met substituted) had approximately 0.4 mm largest dimension and grew in 24-well plates at 4°C in 2-µ1 hanging drops composed of 1/1 24 mM NAG-containing protein solution/crystallization solution (50 mM Na acetate, pH 4.6, 0.2 M Na-malonate and 2% w/v PEG-8K from Hampton Research, USA). The crystals, harvested in crystallization solution supplemented with 25% glycerol, and, in the case of the native NAG-containing crystals, with 2 mM HgCl_2_, (added aiming initially at phasing by anomalous diffraction) were frozen in liquid nitrogen and used for diffraction data collection at 100 K (Oxford Cryosystems) at the ESRF synchrotron (Grenoble). One crystal was soaked with arginine by adding 0.5 µl of 0.2 M L-arginine-hydrochloride to the drop 24 hours before harvesting. Another crystal was freed from NAG by successive three 2-hour passages through 5 µl drops of NAG-free crystallization solution prior to harvesting in the cryobuffer and freezing.

Three crystals of the complete enzyme were used for data collection. Crystal NAGK_1_ belonged to one type and diffracted X-rays at 3.1 Å-resolution. It grew at 4°C in sitting drops composed of 0.4 µl of reductively methylated protein solution without ligands and 0.4 µl crystallization solution containing 0.2 M NH_4_I and 20% PEG3350 (Hampton Research, USA). Crystals NAGK_2_ and NAGK_3_ (3.8 and 3.25 Å- resolution, respectively) were of the same type, and grew in sitting drops at 4°C in the presence of 40 mM NAG using a crystallization solution containing 0.2 M ammonium citrate pH 7.0 and 12% PEG3350 (and 1.5% PEG6000 as an additive for crystal 3). Crystallization solutions with PEG3350 concentration raised to 40% were used as a cryobuffer for liquid nitrogen freezing.

The data for the crystals of the truncated enzyme were processed with MOSFLM [34] or XDS [35], SCALA [36], and TRUNCATE [37] (Table 1). The orthorhombic cell (space group *P*2_1_2_1_2_1_) contained four subunits in the asymmetric unit and 49% solvent content (Table 1). Crystal NAGK_1_ of the complete enzyme had a monoclinic cell (space group *C*2) with two subunits in the asymmetric unit, whereas crystals 2 and 3 had triclinic cells (space group *P*1), with eight subunits in the asymmetric unit.

### Structure determination and refinement

Table 2 summarizes refinement and model data statistics. The initial single-wavelength (0.979 Å) anomalous diffraction (SAD) phases for the Se atoms of the Se-Met AAK crystal diffracting to 3.4 Å resolution, obtained with ShelxC/D/E [38], were improved by refining the Se atom positions with the SAD phasing mode of PHASER [39], implementing density modification, histogram matching, solvent flattening and density averaging with the program DM [40]. A partial model without side chains was built and used successfully as a search model to obtain initial phases by molecular replacement with MOLREP [41] in a 2.2 Å dataset from the NAG-containing crystal. Rigid body refinement was performed stepwise with increasing resolution, followed by restrained refinement using REFMAC [42], alternating with interactive graphic modeling using COOT [43]. The model encompassed all the residues of each subunit, except for the His-tag and four-five N-terminal and C-terminal residues, and it also included one NAG molecule and two Hg atoms per chain sitting next to the S atoms of two Cys residues. B-factors and positional non crystallographic symmetry restraints were used and gradually released as refinement progressed. All the diffraction data were used throughout the refinement process, except for the 5% randomly selected data used for calculating R_free_. TLS (translation/libration/screw) [44] was applied in the final refinement steps with the TLSMD server for the definition of the TLS groups [45]. The structure obtained in this way to 2.2 Å resolution was used as a search model for molecular replacement with MOLREP to obtain phases for the crystals of the truncated enzyme without ligands or soaked with arginine. Refinement for these crystals was carried out as described for the NAG-containing structure (Table 2).

For the complete enzyme, initial phases were obtained for the 3.1 Å dataset collected from the NAGK_1_ crystal by molecular replacement with PHASER using one subunit of the truncated enzyme as search model to yield a dimer in the asymmetric unit. As refinement progressed, electron density became clearer for the DUF619 domain of the two subunits, and a partial model (about 75% of the domain main-chain) was built. This incomplete NAGK_1_ model was then used to obtain an initial solution for the NAGK_2_ crystal by molecular replacement with MOLREP, consisting of 4 dimers forming two tetramers in the asymmetric unit. Further phase improvement was achieved by density averaging between the NAGK crystals 1 and 2, using DMMULTI of the CCP4 suite [46] in conjunction with non crystallographic symmetry averaging, solvent flattening and histogram matching for each crystal. The complete NAGK model was then built for each subunit (except for the 9 initial and the 11 terminal residues) in the two crystals. Refinement could then be carried out until the appropriate quality indicators were obtained for the NAGK_2_ structure (Table 2). However, refinement was not completed for NAGK_1_, which is likely to be due to the presence of some unsolved twinning which was apparent during data collection. Refinement for NAGK_2_ was done by always maintaining tight non crystallographic symmetry restraints and 6 TLS groups per subunit in the final steps. Despite the low resolution (3.8 Å), the quality of the maps allowed the recognition of most of the side chains in the DUF619 domain. The third dataset, collected to 3.25 Å resolution (Table 2) solved by molecular replacement (using the complete subunit of the NAGK_2_ crystal as the model), yielded eight subunits in the asymmetric unit forming two tetramers. The model, including two NAG molecules, was built and refined to 3.25 Å resolution, as for the NAGK_2_ crystal, except for the N-terminal helix of four subunits (two per tetramer at the interdimeric junction), which presented poor density.

The final models for the crystals of the truncated enzyme without NAG, with NAG, and with arginine, and of the complete NAGK_2_ and NAGK_3_ crystals, yielded good R_factor_/R_free_ values and showed good stereochemistry (monitored with PROCHECK) [47]. Superposition of the structures and the r.m.s.d. calculations were carried out with LSQKAB program [48] using default parameters. The buried surface areas and contacts between the subunits were calculated with a probe of 1.4 Å radius using PISA [49]. Subdomain movements were analyzed with DynDom [50]. Figures of protein structures were drawn using PyMOL (http://www.pymol.org).

### Other methods

Thermal stability was assessed with the thermofluor approach [17] in 96-well sealed plates using a real-time PCR system (Applied Biosystems model 7500 Fast), monitoring Sypro Orange (Invitrogen, USA) fluorescence with gradual temperature increase (1°C/min). The 20-µl samples contained 0.2 mg/ml protein in 0.1 M Hepes pH 7.5, 0.15 M NaCl, Sypro Orange (1∶1000 dilution), and 50 mM L-arginine whenever indicated. The unfolded protein fraction was estimated as the ratio between fluorescence increase at a given temperature and the maximal increase observed (complete unfolding). T_0.5_ is the temperature giving a fluorescence increase corresponding to 50% of the maximum increase.

Analytical size exclusion chromatography through Superdex 200 (10/300 column from GE Healthcare) was carried out at 24°C, as reported elsewhere [3], in 50 mM Hepes pH 7.5/0.25 M NaCl. Protein concentration was determined according to Bradford [51] using bovine serum albumin as a standard.

### Accession numbers

The atomic coordinates and structure factors of yNAGK lacking the DUF619 domain, complexed with NAG (PDB ID: 3ZZF), NAG and arginine (PDB ID: 3ZZH), or ligand-free (PDB ID: 3ZZG), as well as those of the complete enzyme (PDB ID: 3ZZI and 4AB7), have been deposited in the Protein Data Bank (www.pdb.org).

## Supporting Information

Figure S1 **Comparison of the dimers of the AAK domain of yNAGK (A) and EcNAGK (B).** In both dimers, the subunit on the left (lighter) is fixed in the same orientation, to highlight the different relative orientation of the other subunit in both NAGKs, in relation to the fixed subunit. The N-terminal “stand” of yNAGK has been omitted for clarity.
(TIF)

Figure S2 **Stereo view of the interactions of one “stand” with its subunit body (both brown) and with the other “stand” (pink) across the interdimeric junction.**
(TIF)

Figure S3 **Experimental information for the DUF619 domain.** (A) Stereo views of the 2F_o_-F_c_ (blue grid) and F_o_-F_c_ (red grid) electron density maps of the complete DUF619 domain (shown as Cα), contoured at 1.0 and 2.5 σ, respectively. (B) Detailed view of a region of the domain, with the model in sticks representation (yellow, blue and red, carbon, nitrogen and oxygen atoms, respectively), to illustrate that the map quality allows identifying most amino acid side chains (labelled for some residues).
(TIF)

Table S1 **Oligonucleotides used in cloning and site-directed mutagenesis.**
(DOC)
