# Supplementary figures and images for: Insight on an Arginine Synthesis Metabolon from the Tetrameric Structure of Yeast Acetylglutamate Kinase
Source: PLoS One. 2012 Apr 18;7(4):e34734. doi: 10.1371/journal.pone.0034734 (PMC3329491; doi:10.1371/journal.pone.0034734)

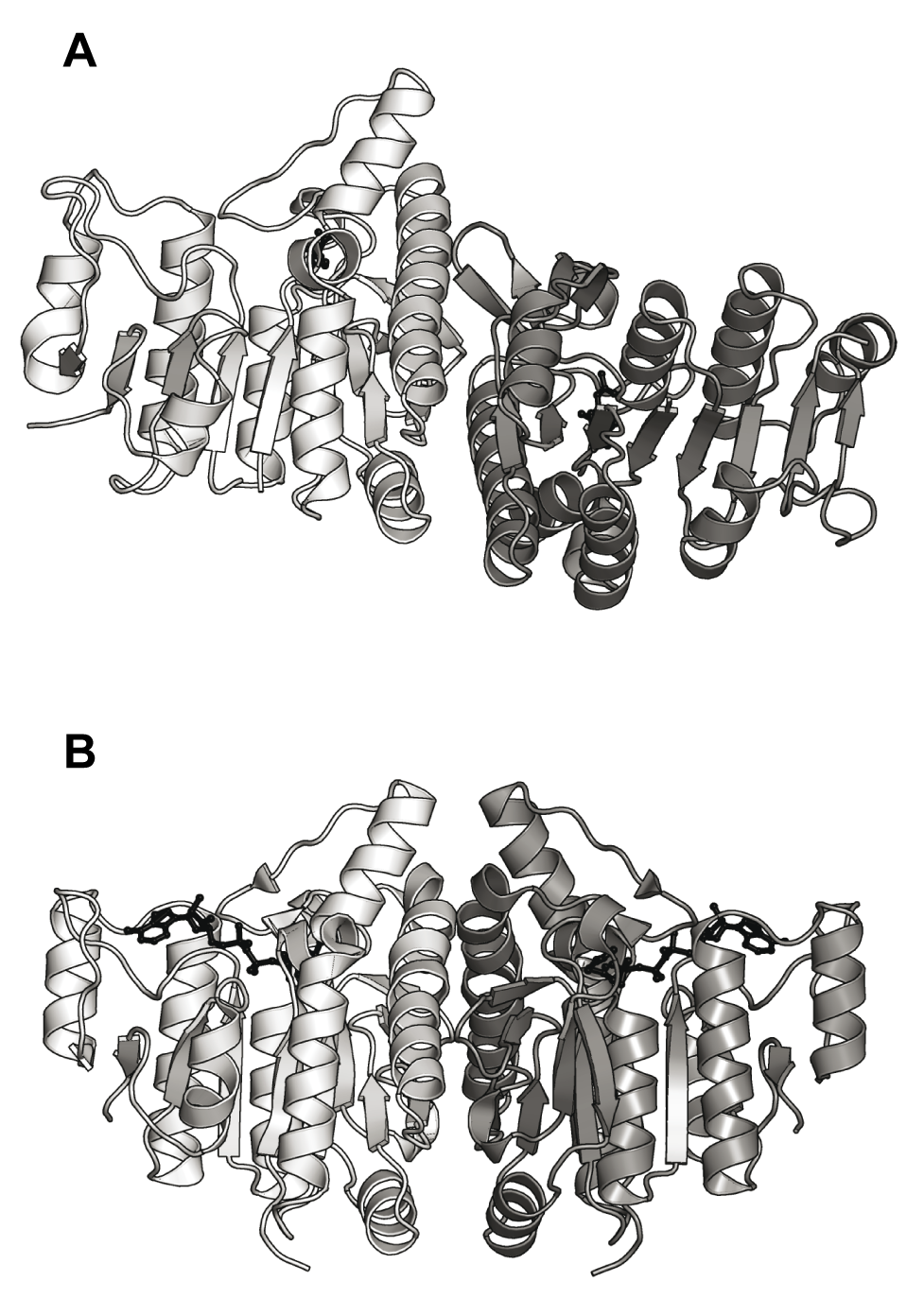

Supplement: Figure S1 — Comparison of the dimers of the AAK domain of yNAGK (A) and EcNAGK (B). In both dimers, the subunit on the left (lighter) is fixed in the same orientation, to highlight the different relative orientation of the other subunit in both NAGKs, in relation to the fixed subunit. The N-terminal “stand” of yNAGK has been omitted for clarity. (TIF) [file pone.0034734.s001.tif]

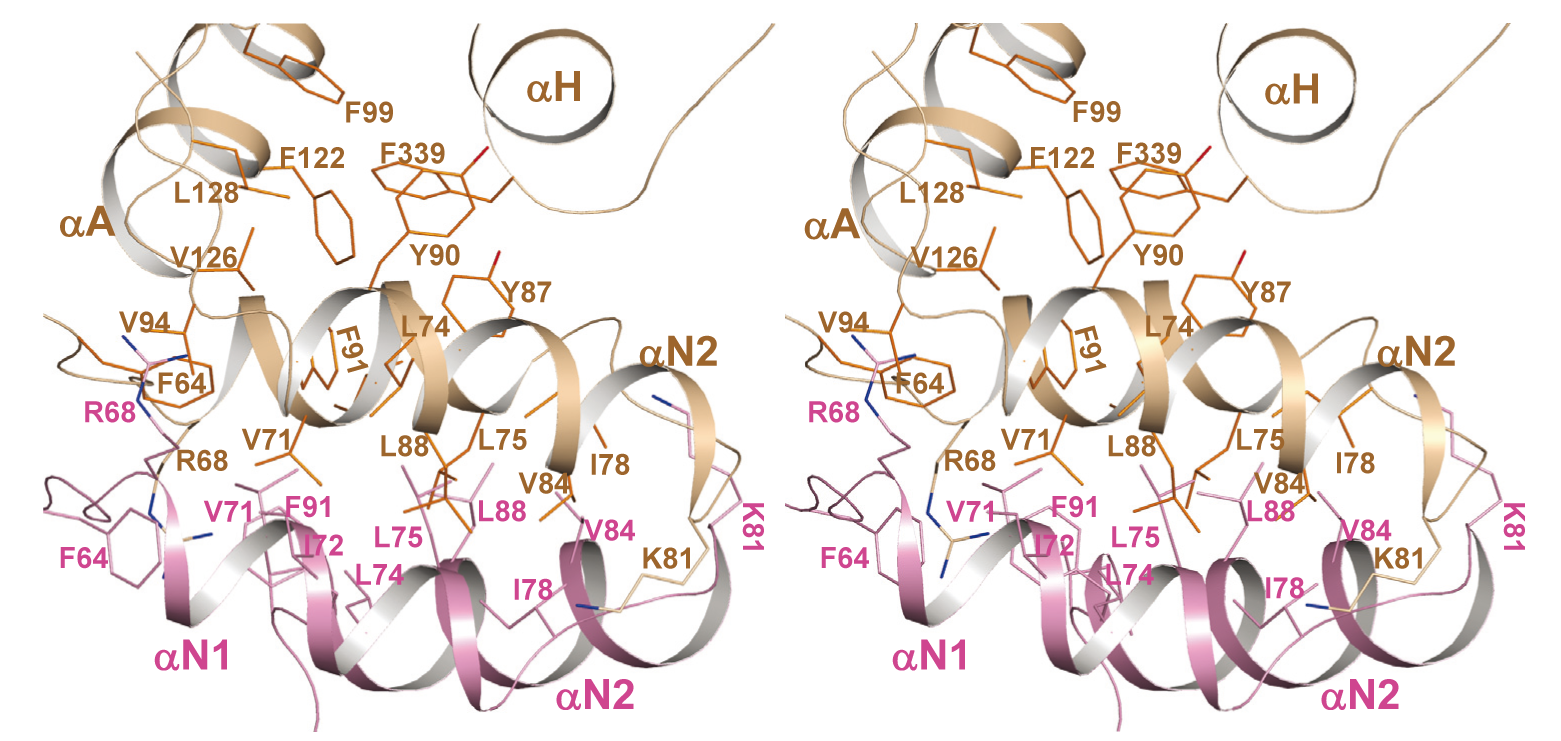

Supplement: Figure S2 — Stereo view of the interactions of one “stand” with its subunit body (both brown) and with the other “stand” (pink) across the interdimeric junction. (TIF) [file pone.0034734.s002.tif]

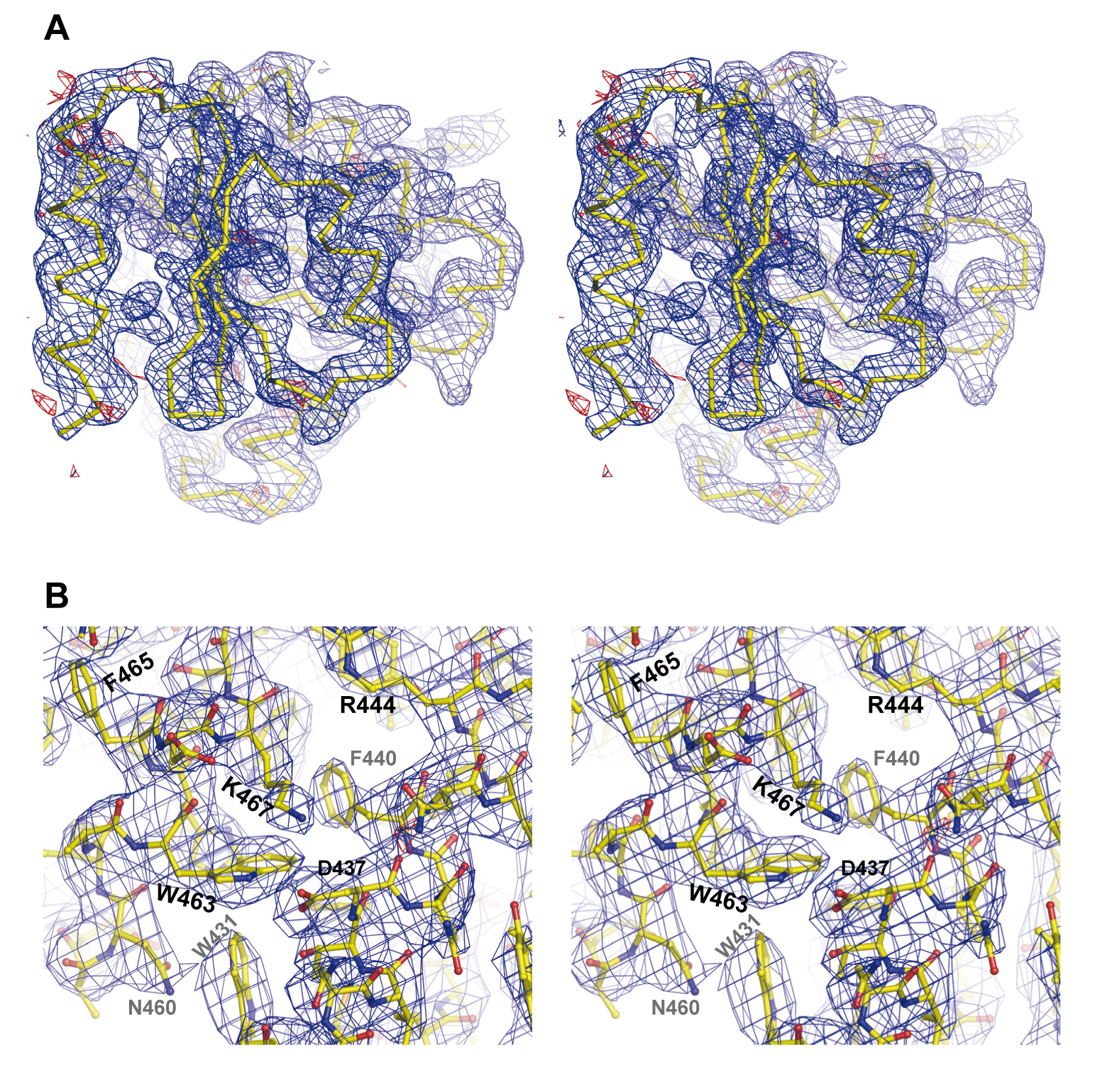

Supplement: Figure S3 — Experimental information for the DUF619 domain. (A) Stereo views of the 2Fo-Fc (blue grid) and Fo-Fc (red grid) electron density maps of the complete DUF619 domain (shown as Cα), contoured at 1.0 and 2.5 σ, respectively. (B) Detailed view of a region of the domain, with the model in sticks representation (yellow, blue and red, carbon, nitrogen and oxygen atoms, respectively), to illustrate that the map quality allows identifying most amino acid side chains (labelled for some residues). (TIF) [file pone.0034734.s003.tif]
