# Supplementary material for: Insight on an Arginine Synthesis Metabolon from the Tetrameric Structure of Yeast Acetylglutamate Kinase
Source: PLoS One. 2012 Apr 18;7(4):e34734. doi: 10.1371/journal.pone.0034734 (PMC3329491; doi:10.1371/journal.pone.0034734)
Supplement: Table S1 — Oligonucleotides used in cloning and site-directed mutagenesis. (DOC) [file pone.0034734.s004.doc]

**Supplementary Online Data.**

**Table S1. Oligonucleotides used in cloning and site-directed mutagenesis**

| **Primer** | **For** | **Direction** | **Sequence** |
| --- | --- | --- | --- |
| 1 | PCR-cloning of complete yNAGK | Forward | ^5'^GGCCATGGGGCATCATCATCATCATCATGTTTCATCTACTAACGGCTTTTCAG^3'^ |
| 2 |  | Reverse | ^5'^GGCCAAGCTTTCAACTACTTGCTGATGAGTTGAGGGTAG^3'^ |
| 3 | Stop codon-introduction to truncate yNAGK | Forward | ^5'^CAAATTAGTGAAGTGATCCTCCATTGGCG^3'^ |
| 4 |  | Reverse | ^5'^CAATGGAGGATCACTTCACTAATTTGTAAC^3'^ |
| 5 | PCR-cloning of GNAT domain | Forward | ^5'^GGGTACTATGATCAGGAGAGCTAGCAAATTAGTGAAG^3'^ |
| 6 |  | Reverse | ^5'^GACGAGGAGTGGATCCAGTTCAGTACGACC^3'^ |
